# Spatiotemporal characterization of cyclooxygenase pathway enzymes during vertebrate embryonic development

**DOI:** 10.1016/j.ydbio.2024.11.009

**Published:** 2024-11-22

**Authors:** Tess A. Leathers, Raneesh Ramarapu, Crystal D. Rogers

**Affiliations:** Department of Anatomy, Physiology, and Cell Biology, University of California, Davis, School of Veterinary Medicine, Davis, CA, USA

**Keywords:** Cyclooxygenase, Prostaglandin G/H synthase, Microsomal prostaglandin E synthase-2, Prostanoid, Embryogenesis, Neurulation

## Abstract

Vertebrate development is regulated by several complex well-characterized morphogen signaling pathways, transcription factors, and structural proteins, but less is known about the enzymatic pathways that regulate early development. We have identified that factors from the inflammation-mediating cyclooxygenase (COX) signaling pathway are expressed at early stages of development in avian embryos. Using *Gallus gallus* (chicken) as a research model, we characterized the spatiotemporal expression of a subset of genes and proteins in the COX pathway during early neural development stages. Specifically, here we show expression patterns of COX-1, COX-2, and microsomal prostaglandin E synthase-2 (mPGES-2) as well as the genes encoding these enzymes (*PTGS1, PTGS2*, and *PTGES-2*). Unique expression patterns of individual players within the COX pathway suggest that they may play non-canonical/non-traditional roles in the embryo compared to their roles in the adult. Future work should examine the function of the COX pathway in tissue specification and morphogenesis and determine if these expression patterns are conserved across species.

## 1. Introduction

Perturbation of the cyclooxygenase (COX) pathway during pregnancy is linked to developmental anomalies, but little is known about its typical localization and function in the embryo (Leathers and Rogers, 2023; Antonucci et al., 2012). Inhibition of COX activity during embryogenesis using nonsteroidal anti-inflammatory drugs increases the risk of neural tube defects (Hernandez et al., 2012), abnormal cardiogenesis (Yoon et al., 2018), craniofacial clefts (Hernandez et al., 2012), and impaired gut innervation (Schill et al., 2016) among other issues (Leathers and Rogers, 2023; Antonucci et al., 2012). Meanwhile, after infection or injury, the COX pathway can be upregulated by multiple cytokines (Hughes et al., 1999; Lacroix and Rivest, 1998; Newton et al., 1996; Kuwano et al., 2004), which are linked to a predisposition for several neuropathologies when activated *in utero* (Jiang et al., 2018). COX pathway signaling has been implicated in uterine implantation (Seo et al., 2014), angiogenesis (Kuwano et al., 2004; Kim et al., 2003; Majumder et al., 2016), formation of the central and enteric nervous systems (Hernandez et al., 2012; Schill et al., 2016), skeletal development (Antonucci et al., 2012), and immune system modulation (Rocca and FitzGerald, 2002). Because of its wide-ranging implications, defining the tissues in which COX pathway factors are expressed in the embryo is a crucial first step in understanding how changes in the pathway will affect development.

The isoenzymes, COX-1 and COX-2, catalyze the first step in the biosynthesis of a variety of inflammation-mediating signaling molecules called prostaglandins (PG) and thromboxanes (TX), collectively known as prostanoids. Specifically, after arachidonic acid is freed from phospholipids by phospholipase A_2_ enzymes (PLA_2_), COX isoenzymes convert the long chain fatty acid into PGG_2_, then reduce PGG_2_ into PGH_2_ (Ricciotti and Fitzgerald, 2011). It is because of this activity that COX isoenzymes are also called prostaglandin-endoperoxide synthases and the official gene names of COX-1 and COX-2 are *PTGS1* and *PTGS2*, respectively. COX-1 is traditionally referred to as the “housekeeping” COX isoform and is broadly expressed in adult human tissues (Zidar et al., 2009). Subcellularly, it is generally associated with the endoplasmic reticulum and nuclear envelope (Williams and DuBois, 1996; Morita et al., 1995). However, studies during embryonic development suggest that *PTGS1* abundance may be more spatiotemporally dynamic than in the adult (Cha et al., 2005, 2006; Wang et al., 2002). In contrast, COX-2 is traditionally seen as an inducible isoenzyme, responding to illness or injury (Williams and DuBois, 1996; Yazaki et al., 2012). However, some studies have found COX-2 to be widely distributed in adult human tissues (Zidar et al., 2009). Like COX-1, COX-2 is localized to the endoplasmic reticulum and nuclear envelope, but while COX-1 is equally distributed, in murine 3T3 cells and human and bovine endothelial cells, COX-2 was twice as concentrated in the nuclear envelope than the endoplasmic reticulum (Morita et al., 1995). *PTGS2* gene expression was found at high levels in fetal rat tissue from gestation days 17–20 but was not detected in embryonic tissues at gestational days 7–13 (Stanfield et al., 2003). In zebrafish embryos, *PTGS2* expression was detected as early as two-somite stage in the anterior neuroectoderm (Cha et al., 2006).

Downstream of COX-1 and COX-2, PGH_2_ is further metabolized by terminal prostanoid synthases like microsomal PGE synthase-2 (mPGES-2). mPGES-2 is one of three PGE synthases responsible for producing PGE_2_ (Park et al., 2006). There are two membrane-associated forms, mPGES-1 and mPGES-2, and a cytosolic form, cPGES (Park et al., 2006). mPGES-2 is encoded by the *PTGES2* gene and functions independently from glutathione, unlike the other PGES enzymes (Park et al., 2006; Hattori, 2005). In HEK293 and BEAS-2B cells, mPGES-2 is first synthesized as a Golgi membrane-associated protein then found in the cytoplasm once its N-terminal hydrophobic domain is removed (Murakami et al., 2003). At the tissue level, mPGES-2 is reported in the brain, heart, skeletal muscle, kidney, and liver of adults (Park et al., 2006). The gene encoding mPGES-1, *PTGES*, is reported to be expressed as early as gastrulation stages (Cha et al., 2006) and blocking its translation prevents normal gastrulation movements in zebrafish (Speirs et al., 2010). However, *PTGES2* expression patterns in the embryo are unknown.

Currently, we lack the spatiotemporal expression data for COX pathway enzymes needed to understand the mechanistic role of this pathway at key embryonic stages. Here, using *in situ* hybridization chain reaction (HCR), immunohistochemistry (IHC), and molecular staining, we visualize the expression of three COX pathway enzymes during neurulation in *Gallus gallus* (chicken) embryos (Hamburger and Hamilton, 1992). We show the expression of *PTGS1*, *PTGS2*, *PTGES2* transcripts, and the proteins encoded by each gene (Table 1) to characterize their tissue-specific localization during neural tube closure and fusion stages. Our results demonstrate that COX pathway enzymes are dynamically and broadly expressed in neurulating amniotic embryos. Additionally, while COX-2 appears to be ubiquitously expressed in all cell types, COX-1 and mPGES-2 have unique tissue and subcellular-specific localization.

## 2. Results

### 2.1. COX pathway gene expression across early developmental stages

**Fig. 1.**
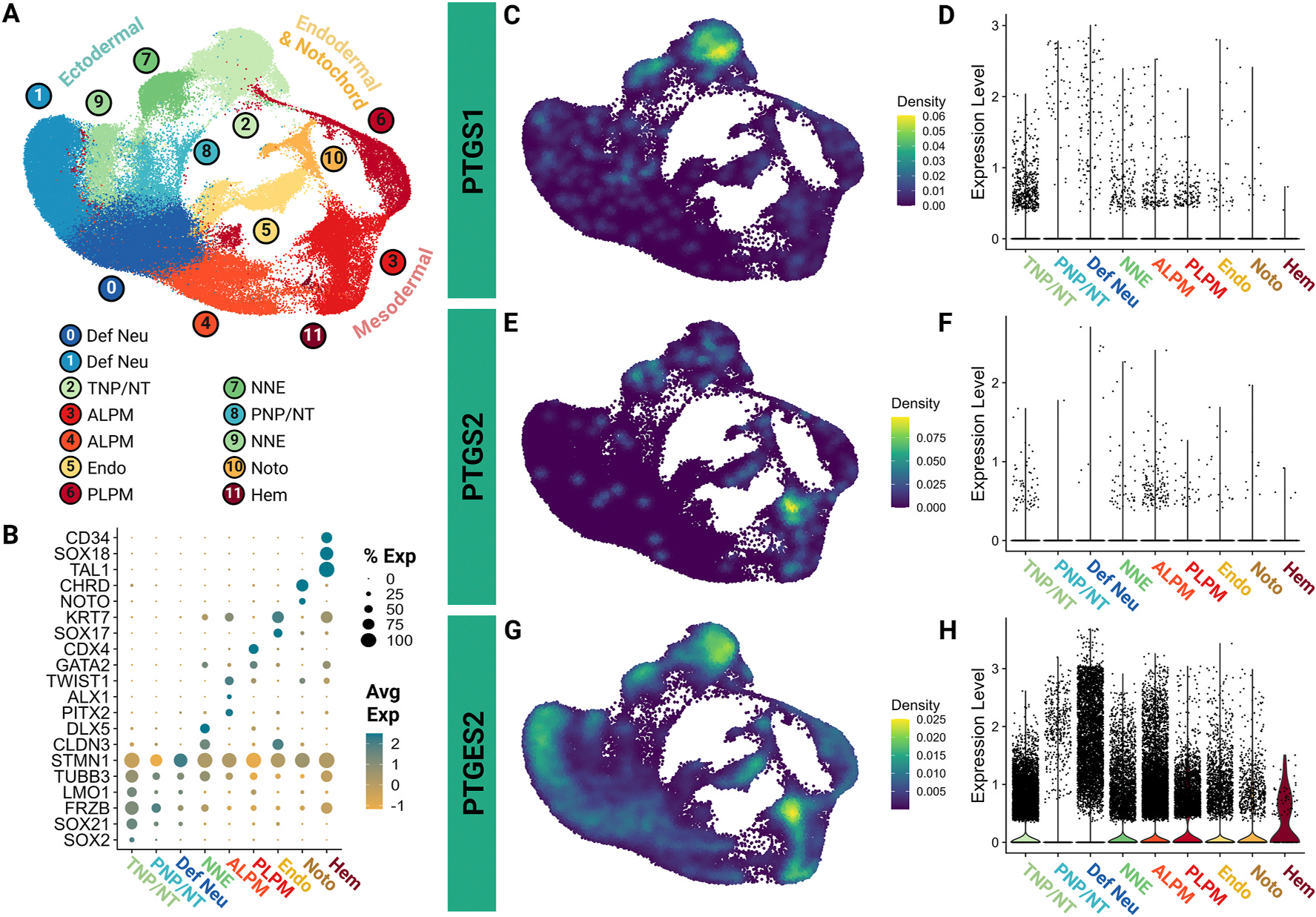
Analysis of publicly available single cell RNA-sequencing data of chick embryos shows cell type-specific expression of select cyclooxygenase pathway isoenzymes. (A) UMAP demonstrating the unsupervised clustering results of single cell RNA-sequencing from whole chick embryos. (B) Dot plot demonstrating cell type-specific marker expression of the 9 major cell types identified across the 12 clusters. (C,E,G) Feature UMAPs showing gene expression of select enzymes by kernel density estimation. (D,F,H) Violin plots demonstrating expression of select enzymes across the 9 major cell types identified. Definitive Neural lineage, Def Neu; Transitional neural plate/neural tube, TNP/NT; Anterior lateral plate mesoderm, ALPM; Endoderm, Endo; Posterior lateral plate mesoderm, PLPM; Non-neural ectoderm, NNE; Posterior neural plate/neural tube, PNP/NT; Notochord, Noto; Hematogenic cells, Hem.

To begin our analysis of COX pathway factor gene expression, we combined two open-source single cell RNA-sequencing datasets spanning chicken stages Hamburger Hamilton (HH) stage 4 - HH9 (Williams et al., 2022; Pajanoja et al., 2023). These stages encompass gastrulation through neural tube closure and neural crest epithelial to mesenchymal transition (EMT) stages. Unsupervised clustering was used to define populations, and these clusters were annotated using published marker genes and gene sets (Fig. 1A and B, Supplemental Fig. 1). Following clustering, we investigated the expression of COX pathway members, including genes encoding phospholipase isoenzymes, COX enzymes, prostanoid terminal synthases, and prostanoid receptors, across the datasets and identified that a majority are expressed at low levels across many tissues. (Fig. 1, Supplemental Fig. 2). Specifically, *PTGS1*, which encodes COX-1, was more highly expressed in the transitional neural plate and neural tube (TNP/NT) and the non-neural ectoderm (NNE) regions (Fig. 1C and D). Over developmental time, the TNP/NT will become the brain and spinal cord while the NNE will become sensory placodes and epidermal tissues. The *PTGS2* gene, which encodes COX-2 protein, was expressed at significantly lower levels across ectodermally-derived tissues but had increased expression in the anterior lateral plate mesoderm (ALPM) cells (Fig. 1E and F). In contrast to either *PTGS1* or *PTGS2*, *PTGES2*, which encodes mPGES-2, has much broader expression than the upstream pathway enzymes. The definitive neural (Def Neu) population had the highest *PTGES2* expression, but the gene was expressed at high levels in all cell types with the exception of the posterior neural plate/neural tube (PNP/NT) (Fig. 1G and H).

### 2.2. COX-1 transcript and protein expression during neurulation

**Fig. 2.**
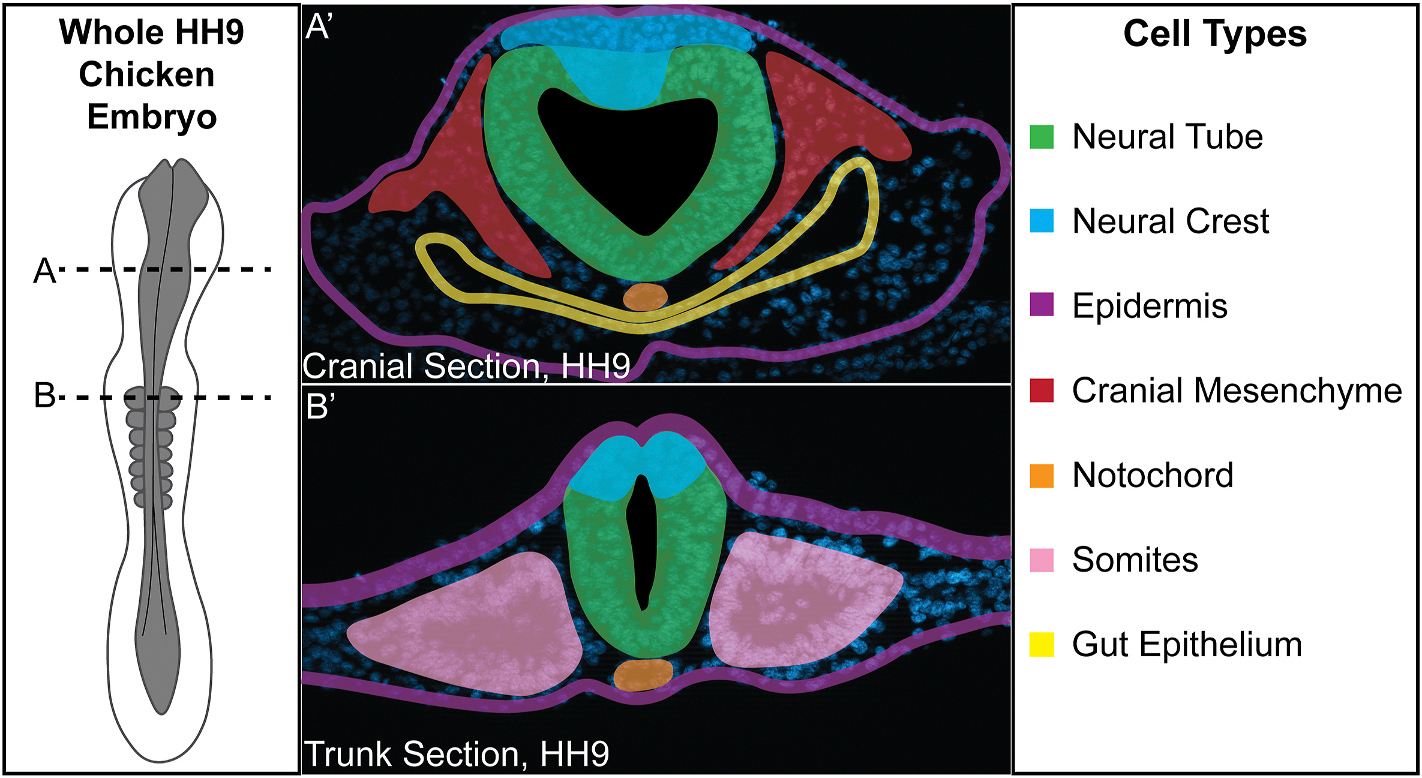
Schematic of cell and tissue types from all three germ layers in a chicken embryo. Whole HH9 chicken embryo illustration shown with transverse cryosections taken at indicated axial levels (A, B). (A′) A cranial transverse section of an HH9 chicken embryo at the axial position indicated by (A) shows cells from the ectodermal (neural crest, neural tube, epidermis), mesodermal (cranial mesenchyme, notochord), and endodermal (gut epithelium) lineages. (B′) A trunk transverse section of an HH9 chicken embryo at the axial position indicated by (B) shows the same cell types in the trunk with the addition of somites from the mesodermal lineage and lack of gut epithelium and cranial mesenchyme due to the more posterior axial level.

**Fig. 3.**
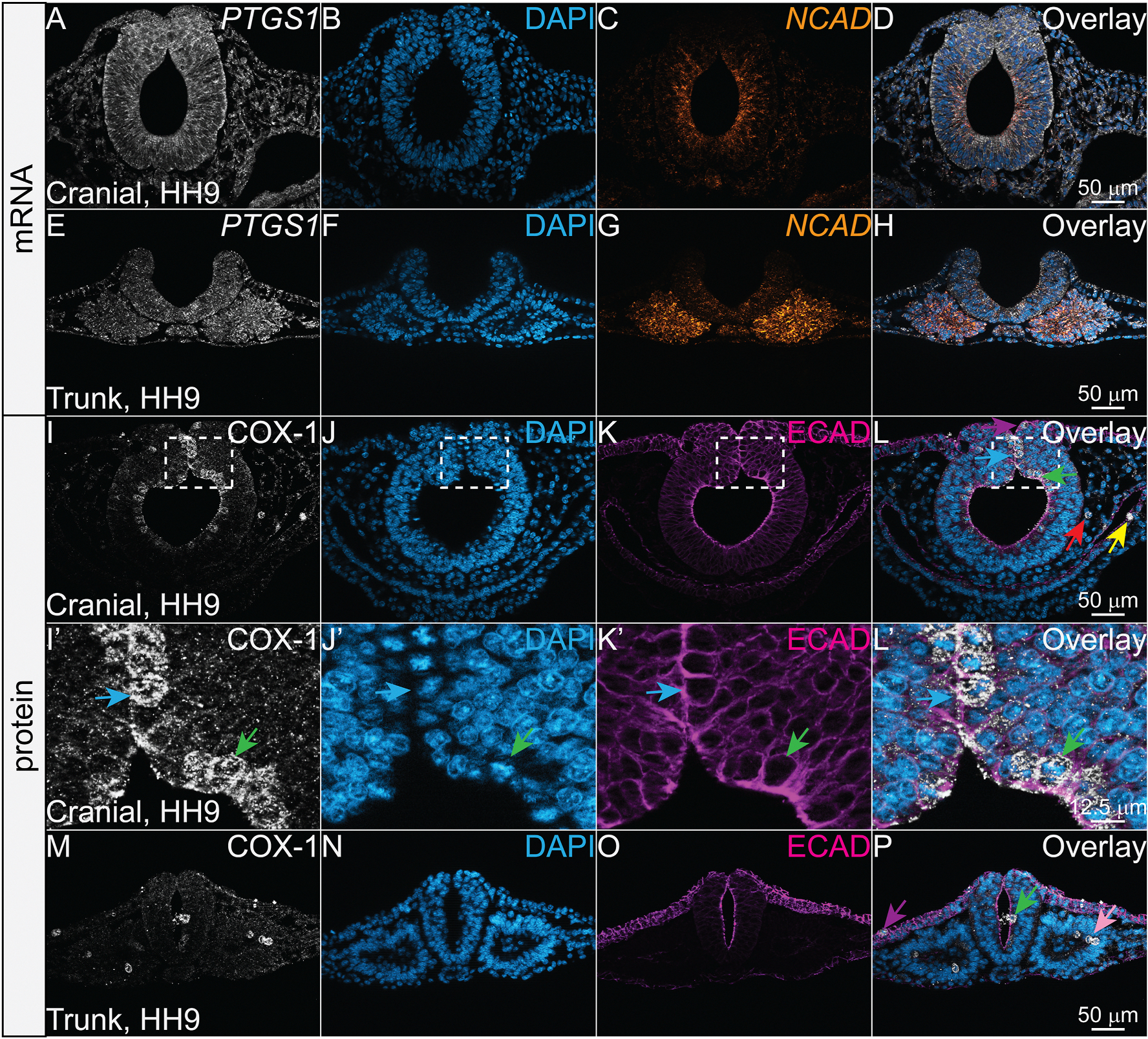
COX-1 is localized to mitotic cells in all three germ layers while its transcript is more broadly expressed. (A–H) Cranial and trunk transverse cryosections of an HH9 chicken embryo with HCR showing COX-1 transcript *PTGS1* (white), the nuclear stain DAPI (blue), neural cadherin transcript *NCAD* (orange), and the overlay of all three channels (D, H). *PTGS1* appears expressed throughout the embryo. (I–P) Cranial and trunk transverse cryosections of an HH9 chicken embryo after IHC using antibodies against the isoenzyme COX-1 (white), the nuclear stain DAPI (blue), and epithelial cadherin, ECAD (magenta). (I′-L′) Zoom in on region outlined in (I–L). (I–P) Arrows indicate COX-1+ mitotic cells with colors coordinating to the cell or tissue types described in Fig. 2. COX-1+ mitotic cells are found in ectodermal derivatives (neural tube in green, neural crest in blue, and epidermis in purple), mesodermal derivatives (cranial mesenchyme in red and somites in pink), and endodermal derivatives (gut epithelium in yellow). Scale bar for each row in the last image of the row.

Previous studies suggested that *PTGS1* is ubiquitously expressed during embryonic stages (Cha et al., 2006; Wang et al., 2002) and becomes more spatially restricted as development progresses (Cha et al., 2005), but *PTGS1* gene and subsequent COX-1 protein spatial expression has not yet been characterized at these early developmental stages in amniotic embryos over the course of their development. To spatially visualize the *PTGS1* gene expression identified in single-cell analysis (Fig. 1C–D) during neurulation, we used HCR in chicken embryos at stages HH7–10 in conjunction with a DAPI stain to visualize nuclei. We provide a color-coded schematic map of HH9 chicken embryo transverse cryosections to delineate specific tissues that express each factor (Fig. 2). We performed HCR for *PTGS1* with neural cadherin (*NCAD*) as a marker of neural tube and paraxial mesoderm because single cell analysis (Fig. 1) suggested that *PTGS1* would be expressed robustly in the neural tube compared to other tissues. We observed that *PTGS1* transcript appears to be expressed ubiquitously throughout the embryo from HH7–10 and that its expression does overlap with *NCAD* in the neural tube and paraxial mesoderm (Fig. 3A–H).

To characterize COX-1 protein expression and localization, we performed IHC using antibodies against COX-1 paired with epithelial cadherin (ECAD), which localizes to epithelial cell membranes and is broadly expressed, and DAPI stain in chicken embryos at stages HH7–10. Fixation methods were optimized specifically for the COX pathway proteins based on recent work showing this is a necessary step in avian embryos (Supplemental Fig. 3) (Echeverria et al., 2025). Notably, COX-1 is more distinctly visualized with 2% TCA fixation compared to 4% PFA fixation (Supplemental Fig. 3) as has been identified for other cytoplasmic and structural proteins (Echeverria et al., 2025). Additionally, the specificity of the COX pathway antibodies used in the study was confirmed via increased antigen-specific signal after overexpression of constructs encoding full-length enzymes (Supplemental Fig. 4). In contrast to the ubiquitous *PTGS1* transcripts, the COX-1 protein appears to be specifically upregulated in cells undergoing mitosis across all axial levels and in cells derived from all three germ layers (Fig. 3I–P, colored arrows match tissues in Fig. 2).

In cells derived from the ectoderm, the COX-1 signal was identified in mitotic cells of the neural tube, neural crest, and epidermis (Fig. 3I–P, green, blue, and purple arrows). During early neural development and prior to cortical histogenesis, nuclei from neuroepithelial cells undergo interkinetic nuclear migration and migrate to the apical side of the neural tube to proliferate (Spear and Erickson, 2012). COX-1-positive cells appear to be specifically localized to the apical side of the neural tube and DAPI staining confirms that these cells are mitotic (Fig. 3I’–3L’, P, green arrows). Similarly, in the mesodermally-derived cells, COX-1 was detected in cranial mesenchyme cells undergoing mitosis (Fig. 3I–L, red arrow). Mesodermally-derived somites also undergo interkinetic nuclear migration while proliferating (Langman and Nelson, 1968), and in sections from the trunk axial level, COX-1 is upregulated in the apical side of the developing somites in cells undergoing mitosis (Fig. 3M–P, pink arrow). In the endodermally-derived cells, COX-1 was detected in mitotic cells lining the developing gut (Fig. 3I–L, yellow arrow). Among the various cell types, COX-1 is expressed in the cytosol of mitotic cells, and is mutually exclusive from DAPI-stained DNA throughout the stages of mitosis (Fig. 3I–P).

**Fig. 4.**
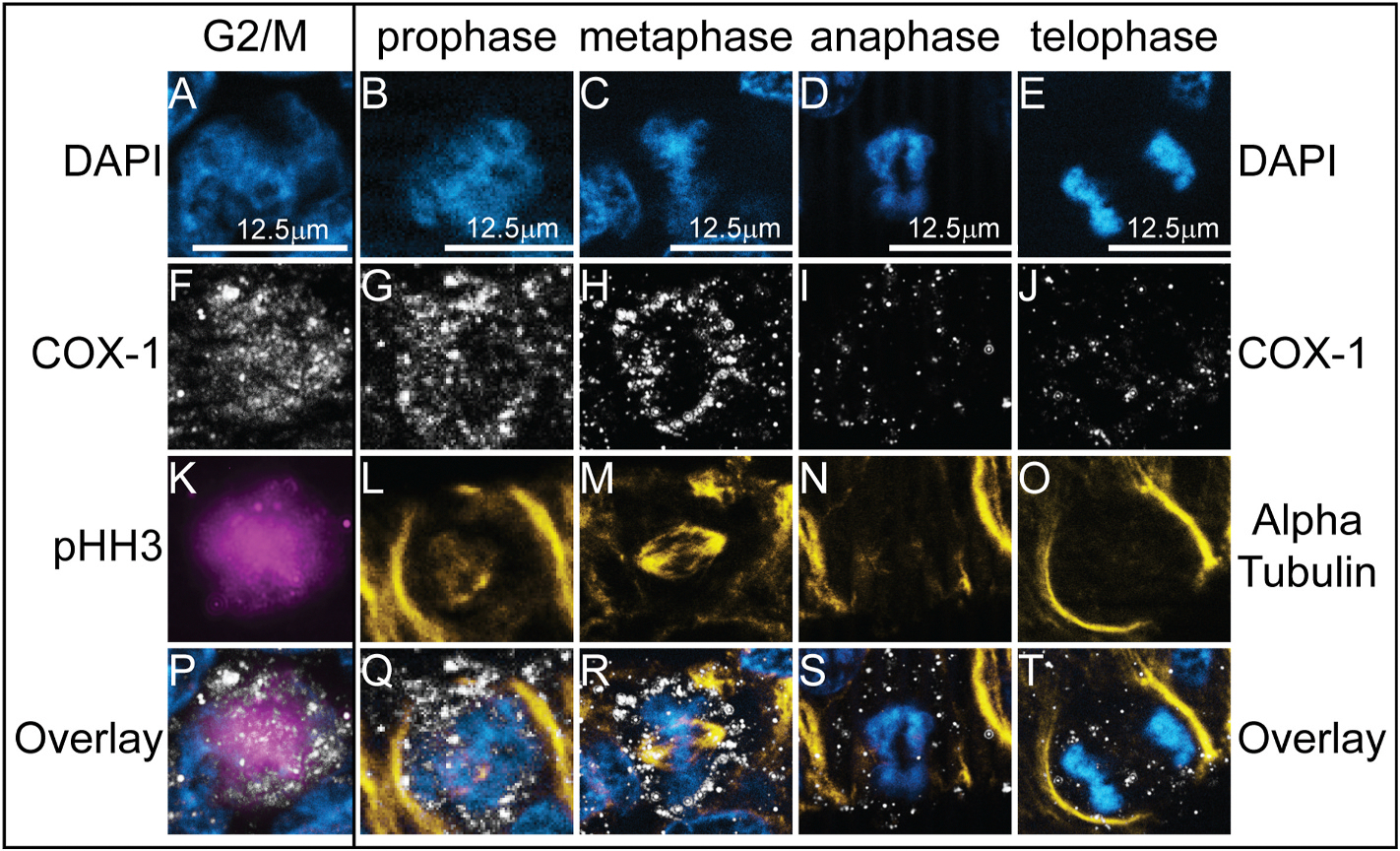
COX-1 protein is upregulated in cells undergoing mitosis in the chicken embryo. IHC with (A–E) the nuclear stain DAPI (blue) and antibodies against (F–J) the isoenzyme COX-1 (white), (L–O) the microtubule subunit Alpha Tubulin (yellow), and (K) the G2/M phase marker phosphohistone H3, pHH3 (magenta) in HH9 and HH10 chicken embryos shows that COX-1 is present in cells during mitosis. Overlays of all three channels shown in (P–T). Scale bar for each column in the first image of the column. Zoom-ins of individual cells taken from images in Supp. Fig. 5.

To characterize the expression and relative localization of COX-1 during each phase of mitosis, we paired IHC of COX-1 with phosphorylated histone H3 (pHH3) to mark the G2 to M transition, alpha tubulin to mark microtubules, and DAPI to mark the chromosomes. COX-1 first appears in cells during G2 and is expressed at lower levels by telophase (compare G2-M, prophase, and metaphase to anaphase and telophase, Fig. 4A–T, Supplemental Fig. 5). COX-1 appears mutually exclusive from the DAPI signal and may localize to the cytoplasm in a perinuclear location (Fig. 4P–T). COX-1 is expressed in 84.3% of cells positive for pHH3 (n = 7 embryos, 2 sections from each). During anaphase, COX-1 protein signal weakens and by the end of telophase, COX-1 signal appears absent or at low levels in non-dividing cells (Fig. 4A–T, Fig. 3I–P).

### 2.3. COX-2 transcript and protein expression during neurulation

**Fig. 5.**
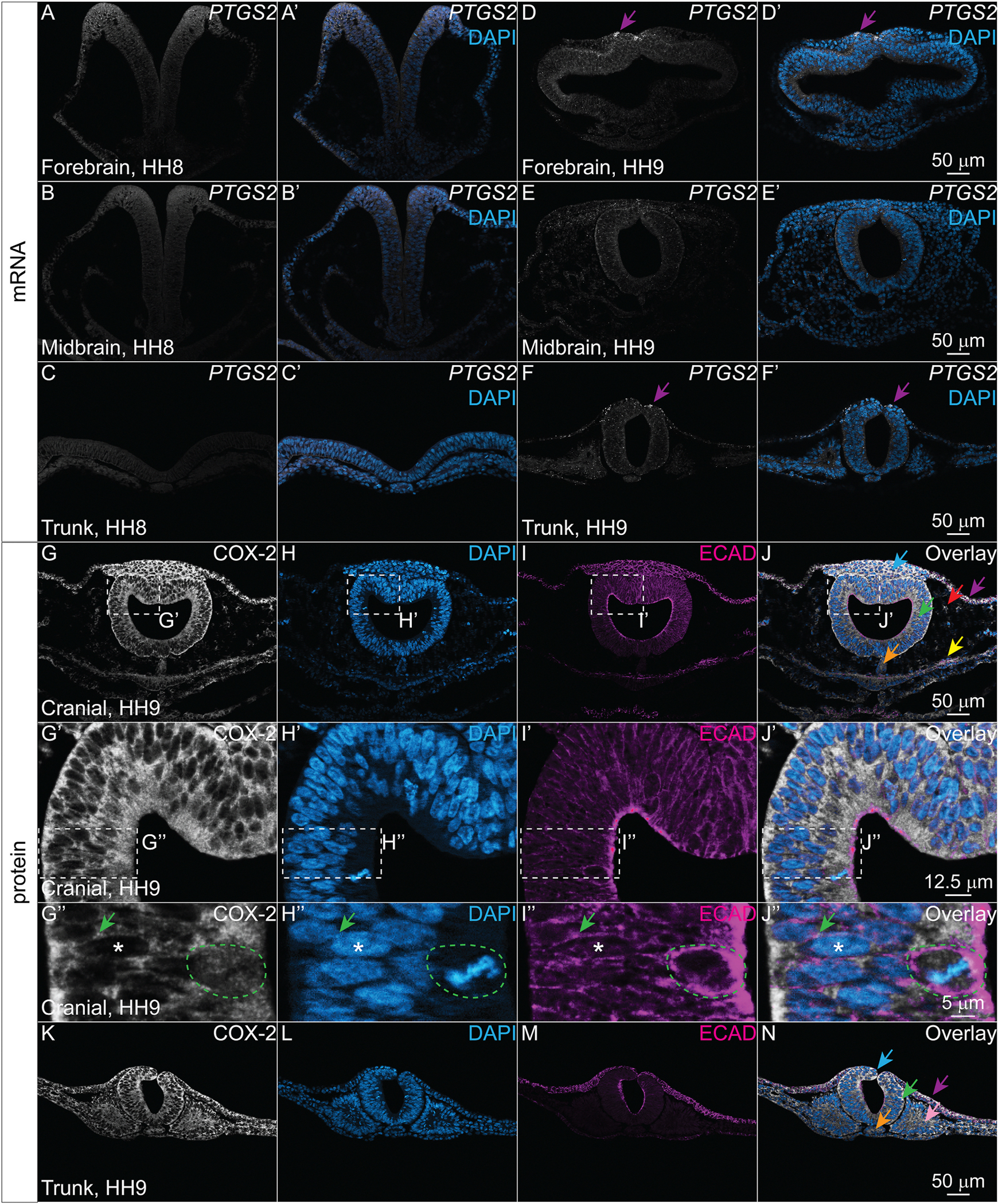
COX-2 is broadly localized throughout the embryo during neurulation but its transcript is expressed at low levels. (A-C′) Transverse cryosections of an HH8 chicken embryo and (D-F′) an HH9 chicken embryo from the forebrain, midbrain, and trunk axial levels with HCR showing COX-2 transcript *PTGS2* (white) and nuclear stain DAPI (blue). (D, D′, F, F′) Purple arrows indicate strong *PTGS2* signal in the dorsal epidermis starting at HH9 in the forebrain and trunk regions (G–N) Cranial and trunk transverse cryosections of an HH9 chicken embryo showing IHC for the isoenzyme COX-2 (white), the nuclear stain DAPI (blue), and membrane-localized ECAD (magenta). (G–N) COX-2 is expressed broadly throughout the embryo. (G”-J″) Within cells, COX-2 signal colocalizes with ECAD (green arrow), is absent from DAPI + nuclei (asterisk), and appears more diffuse in mitotic cells (green outline). Scale bar for each row in the last image of the row.

COX-2 mRNA and protein expression in rat fetal tissues were reported to start at 15 days of gestation (Stanfield et al., 2003), but in zebrafish *Ptgs2* was seen as early as the two-somite stage, when organogenesis is just beginning (Cha et al., 2006; Kimmel et al., 1995). Based on our analysis of avian single cell RNA-sequencing datasets, *PTGS2* expression is relatively low, but existent, in multiple embryonic tissues (Fig. 1E–F). To visualize *PTGS2* transcript expression during neurulation, we performed HCR in chicken embryos at stages HH7–10. We observed that from HH7–8, it is difficult to visualize *PTGS2* signal due to its low transcript levels (Fig. 5A–C’). At HH9, *PTGS2* transcript becomes more distinctly expressed in the forebrain and the trunk epidermis with low levels of expression in the midbrain (Fig. 5D–F’, purple arrows). In the trunk axial level, *PTGS2* expression is apparent in multiple tissues, but it appears most highly expressed in dorsal tissues, particularly at the joining of the neural folds, and in the superficial epidermis (Fig. 5D–F’, purple arrows).

To characterize COX-2 protein expression and localization, we performed IHC for COX-2 with nuclear DAPI stain paired with membrane-localized ECAD in chicken embryos at stages HH7–10. We observed ubiquitous COX-2 protein signal throughout the embryo (Fig. 5G–N). Within cells, COX-2 localized in the cytoplasm and was absent from the nuclei marked by DAPI (Fig. 5G’–5J”, asterisk). In neuroepithelial cells, COX-2 appears to overlap with ECAD in the lateral cell membranes (Fig. 5G”–5J”, green arrow). In dividing cells, where the nuclear membrane has been dissolved, COX-2 appears more diffuse within the cell (Fig. 5G”–5J”, green outline). COX-2 protein expression is similar in the trunk axial levels, with ubiquitous expression across cell types and protein localization to the cytosol (Fig. 5K–N).

### 2.4. mPGES-2 protein and transcript expression and unique subcellular localization during neurulation

**Fig. 6.**
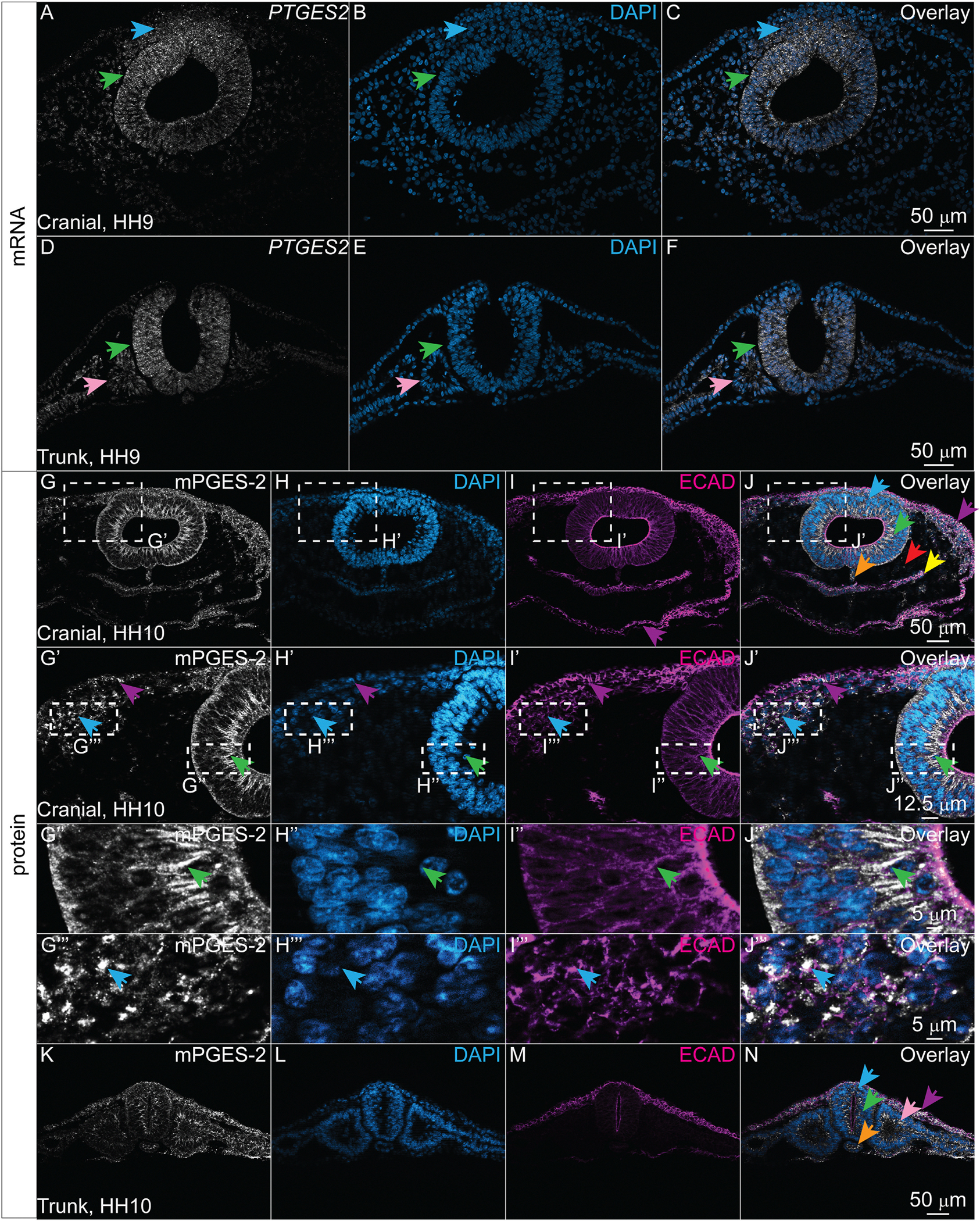
mPGES-2 protein and transcript are present in multiple cell types. (A–F) Cranial and trunk transverse cryosections from an HH9 chicken embryo with HCR showing mPGES-2 transcript *PTGES2* (white) and the nuclear stain DAPI (blue). *PTGES2* signal appears most expressed in the neural tube, neural crest, and somite regions as indicated by green, blue, and pink arrows, respectively. (G–N) Cranial and trunk transverse sections of an HH10 chicken embryo with IHC showing the terminal prostanoid synthase mPGES-2 (white), nuclear stain DAPI (blue), and ECAD (magenta). (G–J) mPGES-2 is broadly expressed across multiple tissues. (G″-J″) Zoom in of region outlined in (G′-J′) reveals that mPGES-2 co-localizes with ECAD in the membrane of neuroepithelial cells of the neural tube (green arrow), while (G‴-J‴) in epidermal and neural crest cells, mPGES-2 is found in punctate compartments mutually exclusive from ECAD (purple and blue arrows, respectively). (G″-J″ and G‴-J‴) Zoom ins of G′-J′ in the neural tube and neural crest cells, respectively. Scale bar for each row in the last image of the row.

Single cell RNA-sequencing data showed that across all developmental stages included, the transcript encoding the terminal prostanoid synthase mPGES-2, *PTGES2*, was expressed widely across multiple tissues (Fig. 1G–H). mPGES-2 acts downstream of the COX isoenzymes to convert PGH_2_ into PGE_2_ (Ricciotti and Fitzgerald, 2011). To date, no studies have characterized the spatial expression profiles of mPGES-2 or its corresponding gene *PTGES2* in the developing embryo. To characterize *PTGES2* expression in vertebrate embryos during neurulation, we used HCR in chicken embryos at stages HH7–10. We observed *PTGES2* expression in all tissues, but it appeared strongest in the neural tube, neural crest, and mesodermally-derived tissues including the somites (Fig. 6A–F, green, blue, and pink arrows).

To characterize mPGES-2 protein expression and localization during neurulation we performed IHC for mPGES-2 with DAPI nuclear stain and ECAD in chicken embryos at stages HH7–10. We observed mPGES-2 protein signal throughout the embryo across multiple cell and tissue types (Fig. 6G–N). In neuroepithelial cells, mPGES-2 co-localized with ECAD at the cell membrane (Fig. 6G”–6J”, green arrow). In contrast, mPGES-2 appeared localized to punctate condensates within epidermal and neural crest cells (Fig. 6G’–6J′, 6G‴–6J‴, purple and blue arrows, respectively). The protein was also detected in trunk axial level tissues and appeared to localize to the membrane with ECAD in epithelial epidermis, neural tube, and somites, but also showed punctate localization in a subset of cells (Fig. 6K–N). Of note, mPGES-2 is more distinctly visualized with 2% TCA fixation compared to 4% PFA fixation as observed with COX-1 (Supplemental Fig. 3).

## 3. Discussion

According to the classical understanding of cyclooxygenases, COX-1 is the constitutive isoenzyme and COX-2 is the inducible isoenzyme (Yazaki et al., 2012). This understanding appeared consistent at the transcript level in chicken embryos, where *PTGS1* mRNA appeared largely ubiquitous while *PTGS2* mRNA was spatiotemporally restricted (Figs. 1, 3 and 5). However, visualizing the corresponding protein localization demonstrated the importance of characterizing expression at both the gene and protein level.

Despite its broad gene expression, COX-1 protein signal was only detectable in mitotic cells (Figs. 3 and 4). This stark contrast between mRNA and protein expression suggests that COX-1 may be post-transcriptionally regulated to allow for dynamic changes in protein abundance depending on the cellular context. Past studies demonstrated that COX-1 protein is degraded by the ubiquitin-proteasome system within 10 min of intracellular calcium influx in human megakaryocytic MEG-01 cells (Yazaki et al., 2012). Cell cycle progression is also regulated by intracellular calcium, with levels peaking at anaphase (Wilding, 1996; Tombes and Borisy, 1995), which correlates with the reduction of COX-1 that we see in our mitotic cells. Our results suggest that in developing embryos, COX-1 expression may be post-transcriptionally or post-translationally regulated depending on the cellular context and environment (Fig. 4).

The specific localization of COX-1 to mitotic cells suggests that COX-1 may play a role in cell division. Past work identified that exposure to COX-inhibiting NSAIDs prevents cell division *in vitro* in multiple cancer cell lines (Diederich et al., 2010; Kundu et al., 2002) and COX-2 inhibition downregulates expression of genes associated with the spindle assembly checkpoint (Bieniek et al., 2014). Further, the signals and receptors downstream of COX-1 and COX-2 are linked with cell proliferation (Mo et al., 2015; Jiang and Dingledine, 2013; Jimenez et al., 1975). Recent work from our lab showed that exposure to the COX-1 and COX-2-inhibiting non-steroidal anti-inflammatory drug (NSAID), naproxen, alters the development of ectodermally-derived tissues in axolotl embryos suggesting that they may be necessary for the growth or maintenance of these tissues (Marshall et al., 2024). Future research should investigate the role of COX isoenzymes, and particularly COX-1, in cell division during embryogenesis.

In contrast to COX-1, the *PTGS2* gene was expressed sporadically at low levels and COX-2 protein was detected ubiquitously throughout embryonic cell types during neurulation stages (Figs. 1 and 4). This break from previously described COX expression patterns may be attributed to the unique cellular context of embryonic development. During embryogenesis, drastic morphological changes occur on a cellular and tissue level. For example, neural crest cells undergo EMT, delaminating from the neuroepithelial cells and migrating dorsolaterally out of the neural tube upon its fusion (Leathers and Rogers, 2022). While processes like EMT are normal and necessary in the embryo, they would represent a disease state in the adult. In fact, COX-2 is upregulated in several cancers (Singh et al., 2005; Zha et al., 2004) and drives cancer cell EMT and invasion (Majumder et al., 2016; Neil et al., 2008; Qiu et al., 2014).

Downstream of the COX isoenzymes, we characterized the expression of the terminal prostanoid synthase, mPGES-2. mPGES-2 functionally couples with both COX-1 and COX-2 to synthesize PGE_2_ (Ricciotti and Fitzgerald, 2011). By synthesizing PGE_2_, mPGES-2 can have widespread effects as PGE_2_ is the most abundant PG in the adult and plays both homeostatic, pro-inflammatory, and anti-inflammatory roles depending on the context (Park et al., 2006). Notably, PGE_2_ is linked to ovulation, cardiogenesis, and neural crest development based on prior research (reviewed in Leathers and Rogers, 2023). In the neurulating chicken embryo, the *PTGES2* transcript was expressed broadly with increased signal in the neural tube and neural crest cells. The protein, mPGES-2, appeared to be expressed in all tissues that we visualized (Fig. 6), which would theoretically allow it to act in conjunction with both ubiquitous COX-2 and spatially restricted COX-1 enzymes. Within cells, mPGES-2 varied in subcellular localization (Fig. 6). It is reported to be synthesized as a Golgi membrane-associated protein, which then localizes to the cytosol in its mature form (Ricciotti and Fitzgerald, 2011; Park et al., 2006). The varied subcellular localizations observed in different cell types could represent mPGES-2 in its various maturity states and their associated localizations (Fig. 6). In addition, the dynamic subcellular localization of mPGES-2 was linked to different cell types. In neuroepithelial cells, the mPGES-2 signal appeared in the cell membrane and co-localized with ECAD, but in the collectively migrating and mesenchymal migratory neural crest cells, mPGES-2 appeared to be localized to either subcellular compartments, vesicles, or condensates. Future work will focus on identifying how mPGES-2 subcellular localization may affect its function, specifically if membrane-localized mPGES-2 facilitates cell adhesion or morphological changes (e.g., neural tube closure) and if compartmentalization is necessary for neural crest migration.

In this study we have characterized the spatiotemporal expression of COX pathway enzymes COX-1, COX-2, and mPGES-2 in neurulating avian embryos. Notably, we found that the expression and localization of COX isoenzymes may not fit into previously defined expression patterns from disease cells and adult tissues in the embryo. The dynamic differences observed between transcript and protein signal highlights the need to characterize expression at both the gene and protein level to understand better when and where factors may be functioning in developmental processes. Future work will focus on defining the role of these COX pathway enzymes and the signals they produce during embryonic development.

## 4. Methods

### 4.1. Chicken embryos

Fertilized chicken eggs were obtained from the Hopkins Avian Facility at the University of California, Davis and incubated at 37 °C to the desired stages according to the criteria of Hamburger and Hamilton (HH). Use and experiments on embryos was approved by the University of California, Davis.

### 4.2. Single cell analysis

COX pathway members in the *Gallus gallus* genome were identified using Ensembl Release 112. Expression profiles for each gene listed in Supp. Table 1 are shown in Supp. Fig. 2. Filtered features matrices for embryos were obtained from peer-reviewed publicly available single cell datasets (NCBI GSE181577 (Williams et al., 2022) and GSE221188 (Pajanoja et al., 2023)). Analyses were performed using Seurat V5 (Satija et al., 2015). Filtered feature matrices were independently log-normalized and scaled. Objects were integrated by sample using the harmony package (Korsunsky et al., 2019) following which dimensionality reduction (determined using knee identification in elbow plot), k-means clustering (resolution determined using clustree package (Zappia and Oshlack, 2018)) and neighbor identification was performed. The embeddings were utilized for Uniform Manifold Approximation and Projection (UMAP) plotting. Cell type annotations for clusters were performed using marker expression from the source manuscripts (Williams et al., 2022; Pajanoja et al., 2023). Kernel density gene expression plots were created using the Nebulosa package (Alquicira-Hernandez and Powell, 2021) and violin plots using the Seurat native VlnPlot function. Figures were organized in BioRender.

### 4.3. Wholemount in situ hybridization chain reaction

Wholemount *in situ* hybridization chain reaction (HCR) was performed using the protocol suggested by Molecular Technologies with minor modifications. Chicken embryos were fixed in 4% paraformaldehyde in phosphate buffer (4% PFA) for 1 h at room temperature (RT), washed in 1X PBS with 0.1% Triton (PBST), and were dehydrated in a series of 25%, 50%, 75%, and 100% methanol. Embryos were stored at − 20 °C prior to beginning HCR protocol. Embryos were rehydrated in a series of 25%, 50%, 75%, and 100% PBST when beginning the HCR procedure, but were not incubated with proteinase-K as suggested by the protocol. Embryos were incubated with 2.5–10μL of probes dissolved in hybridization buffer overnight (12–24 h) at 37 °C. After washes on the second day, embryos were incubated with 10uL each of hairpins H1 and H2 diluted in amplification buffer at RT overnight (12–24 h). Embryos were subsequently incubated with 1:500 DAPI in PBST for 1 h at RT, postfixed in 4% PFA for 1 h at RT or 4 °C overnight (12–24 h), then washed in 1X PBS with 0.1% Tween-20 (PTween). After HCR, all embryos were imaged in both whole mount and transverse section (after embedding in gelatin and cryosectioning frozen samples) using a Zeiss Imager M2 with Apotome capability and Zen optical processing software.

### 4.4. Immunohistochemistry

Immunohistochemistry (IHC) was performed as described previously (Echeverria et al., 2025) and antibodies used in study are listed in Table 2. Briefly, for IHC, chicken embryos were fixed on filter paper in 4% PFA for 15–20 min at RT or in 2% trichloroacetic acid (TCA) for 1 h at RT. After fixation, embryos were washed in 1X TBS (500 mM Tris-HCl, pH 7.4, 1.5 M NaCl, and 10 mM CaCl_2_) containing 0.1% Triton X-100 (TBST + Ca^2+^). For blocking, embryos were incubated in TBST + Ca^2+^ with 10% donkey serum (blocking buffer) for 1 h at RT or overnight (12–24 h) at 4 °C. Primary antibodies were diluted in blocking buffer at indicated dilutions and incubated with embryos for 48–96 h at 4 °C. After incubation with primary antibodies, whole embryos were washed in TBST + Ca^2+^, then incubated with AlexaFluor secondary antibodies diluted in blocking buffer (1:500) overnight (12–24 h) at 4 °C. They were then washed in TBST + Ca^2+^ and 4% PFA-fixed embryos were post-fixed in 4% PFA for 1 h at RT. Antibodies used in the study (Table 2): Rabbit IgG α-COX-1 (Cayman Chemical 160109), Rabbit IgG α-COX-2 (Cayman Chemical 160126), Rabbit IgG α-mPGES-2 (Cayman Chemical 160145), Mouse IgG2a α-ECAD (BD Transduction Laboratories, 61081), Mouse IgG1 α-Alpha Tubulin (Invitrogen, 322588), and Rat IgG2a α-pHH3 (EMD Millipore, MABE939). DNA constructs encoding full-length COX-1, COX-2, and mPGES-2 were unilaterally injected and subsequently electroporated into gastrula stage chicken embryos and the corresponding increase in IHC signal shows antibody specificity (Supp. Fig. 4). After IHC, all embryos were imaged in both whole mount and transverse section (after embedding in gelatin and cryosectioning frozen samples) using a Zeiss Imager M2 with Apotome capability and Zen optical processing software.

## Supplementary Material

Supplemental figures

## Figures and Tables

**Table 1:** COX pathway enzymes characterized in this study.

Protein Name	Gene Name	NCBI Reference Sequence	Aliases

Cyclooxygenase-1 (COX-1)	*PTGS1*	XM_040685541.2	Prostaglandin-Endoperoxide Synthase-1, Prostaglandin G/H Synthase-1, Prostaglandin H*2* Synthase-1
Cyclooxygenase-2 (COX-2)	*PTGS2*	NM_001167719.2	Prostaglandin-Endoperoxide Synthase-2, Prostaglandin G/H Synthase-2, Prostaglandin H*2* Synthase-2
Microsomal Prostaglandin E Synthase-2 (mPGES-2)	*PTGES2*	XM_040685892.2	Membrane-Associated Prostaglandin E Synthase-2

**Table 2:** Antibodies used in the study.

Antibody target	Dilution	Species Reactivity	Isotype/Host	Immunogen	Source	Optimal Fixation (see Supp. Fig. 3)

Cyclooxygenase-1 (COX-1)	1:200	Mouse, ovine	Rabbit IgG	Peptide from the internal region of mouse COX-1	Cayman Chemical 160109,	1h TCA
Cyclooxygenase-2 (COX-2)	1:200	Human, macaque monkey, mouse, ovine, rat	Rabbit IgG	Synthetic peptide corresponding to the C-terminal region of mouse COX-2	Cayman Chemical 160126,	20m PFA
Microsomal Prostaglandin E Synthase-2 (mPGES-2)	1:200	Human, African green monkey, bovine, mouse, ovine, rat	Rabbit IgG	Synthetic peptide from the internal region of human mPGES-2	Cayman Chemical 160145,	1h TCA
Alpha Tubulin	1:200	Human, Mouse, Rat, Sea urchin	Mouse IgG1	Sarkosyl-resistant filament from sea urchin sperm axonemes	Invitrogen, 322588	20m PFA or 1h TCA
Epithelial Cadherin (ECAD)	1:500	Human, Mouse, Rat, Dog	Mouse IgG2a	Amino acids 735–883	BD Transduction Laboratories, 61081	20m PFA or 1h TCA
Phosphohistone H3 (pHH3)	1:500	Human	Rat IgG2a	KLH-conjugated linear peptide corresponding to human Histone H3 (Ser10)	EMD Millipore, MABE939	20m PFA or 1h TCA

## Data Availability

Data will be made available on request.

## References

[R1] Alquicira-Hernandez J, Powell JE, 2021. Nebulosa recovers single-cell gene expression signals by kernel density estimation. Bioinformatics 37, 2485–2487. 10.1093/bioinformatics/btab003. 33459785

[R2] Antonucci R, Zaffanello M, Puxeddu E, Porcella A, Cuzzolin L, Dolores Pilloni Maria, Fanos Vassilios, 2012. Use of non-steroidal anti-inflammatory drugs in pregnancy: impact on the fetus and newborn. Curr. Drug Metabol. 13 (4), 474–490. 10.2174/138920012800166607. 22299823

[R3] Bieniek J, Childress C, Swatski MD, Yang W, 2014. COX-2 inhibitors arrest prostate cancer cell cycle progression by down-regulation of kinetochore/centromere proteins. Prostate 74, 999–1011. 10.1002/pros.22815. 24802614

[R4] Cha YI, Kim SH, Solnica-Krezel L, DuBois RN, 2005. Cyclooxygenase-1 signaling is required for vascular tube formation during development. Dev. Biol. 282, 274–283. 10.1016/j.ydbio.2005.03.014. 15936346

[R5] Cha YI, Kim SH, Sepich D, Gregory Buchanan F, Solnica-Krezel L, DuBois RN, 2006. Cyclooxygenase-1-derived PGE2 promotes cell motility via the G-protein-coupled EP4 receptor during vertebrate gastrulation. Genes Dev. 20, 77–86. 10.1101/gad.1374506. 16391234 PMC1356102

[R6] Diederich M, Sobolewski C, Cerella C, Dicato M, Ghibelli L, 2010. The role of cyclooxygenase-2 in cell proliferation and cell death in human malignancies. Int J Cell Biol 2010. 10.1155/2010/215158. PMC2841246 20339581

[R7] Echeverria CV, Leathers TA, Rogers CD, 2025. Comparative analysis of fixation techniques for signal detection in avian embryos. Dev. Biol. 517, 13–23. 10.1016/j.ydbio.2024.09.002. 39245159 PMC11631674

[R8] Hamburger V, Hamilton HL, 1992. A series of normal stages in the development of the chick embryo. Dev. Dynam. 195, 231–272. 10.1002/aja.1001950404. 1304821

[R9] Hattori M, 2005. Finishing the euchromatic sequence of the human genome. Tanpakushitsu Kakusan Koso 50, 162–168. 15704464

[R10] Hernandez RK, Werler MM, Romitti P, Sun L, Anderka M, 2012. Nonsteroidal antiinflammatory drug use among women and the risk of birth defects. Am. J. Obstet. Gynecol. 206, 228.e1–228.e8. 10.1016/j.ajog.2011.11.019. PMC5893141 22196851

[R11] Hughes FJ, Buttery LDK, Hukkanen MVJ, O’Donnell A, Maclouf J, Polak JM, 1999. Cytokine-induced prostaglandin E2 synthesis and cyclooxygenase-2 activity are regulated both by a nitric oxide-dependent and -independent mechanism in rat osteoblasts in vitro. J. Biol. Chem. 274, 1776–1782. 10.1074/jbc.274.3.1776. 9880560

[R12] Jiang J, Dingledine R, 2013. Role of prostaglandin receptor EP2 in the regulations of cancer cell proliferation, invasion, and inflammation. J. Pharmacol. Exp. Therapeut. 344, 360–367. 10.1124/jpet.112.200444. PMC3558819 23192657

[R13] Jiang NM, Cowan M, Moonah SN, Petri WA, 2018. The impact of systemic inflammation on neurodevelopment. Trends Mol. Med. 24, 794–804. 10.1016/j.molmed.2018.06.008. 30006148 PMC6110951

[R14] Jimenez De Asua L., Clingan D, Rudland PS, 1975. Initiation of cell proliferation in cultured mouse fibroblasts by prostaglandin F2(α). Proc. Natl. Acad. Sci. U.S.A. 72, 2724–2728. 10.1073/pnas.72.7.2724. 170616 PMC432843

[R15] Kim MH, Seo SS, Song YS, Kang DH, Park IA, Kang SB, Lee HP, 2003. Expression of cyclooxygenase-1 and −2 associated with expression of VEGF in primary cervical cancer and at metastatic lymph nodes. Gynecol. Oncol. 90, 83–90. 10.1016/S0090-8258(03)00224-5. 12821346

[R16] Kimmel CB, Ballard WW, Kimmel SR, Ullmann B, Schilling TF, 1995. Stages of embryonic development of the zebrafish. Dev. Dynam. 203, 253–310. 10.1002/aja.1002030302. 8589427

[R17] Korsunsky I, Millard N, Fan J, Slowikowski K, Zhang F, Wei K, Baglaenko Y, Brenner M, Loh P ru, Raychaudhuri S, 2019. Fast, sensitive and accurate integration of single-cell data with Harmony. Nat. Methods 16, 1289–1296. 10.1038/s41592-019-0619-0. 31740819 PMC6884693

[R18] Kundu N, Smyth MJ, Samsel L, Fulton AM, 2002. Cyclooxygenase inhibitors block cell growth, increase ceramide and inhibit cell cycle. Breast Cancer Res. Treat. 76, 57–64. 10.1023/A:1020224503335. 12408376

[R19] Kuwano T, Nakao S, Yamamoto H, Tsuneyoshi M, Yamamoto T, Kuwano M, Ono M, 2004. Cyclooxygenase 2 is a key enzyme for inflammatory cytokine-induced angiogenesis. Faseb. J. 18, 300–310. 10.1096/fj.03-0473com. 14769824

[R20] Lacroix S, Rivest S, 1998. Effect of acute systemic inflammatory response and cytokines on the transcription of the genes encoding cyclooxygenase enzymes (COX-1 and COX-2) in the rat brain. J. Neurochem. 70, 452–466. 10.1046/j.1471-4159.1998.70020452.x. 9453538

[R21] Langman J, Nelson GR, 1968. A radioautographic study of the development of the somite in the chick embryo. J. Embryol. Exp. Morphol. 19, 217–226. 10.1242/dev.19.2.217. 5656455

[R22] Leathers TA, Rogers CD, 2022. Time to go: neural crest cell epithelial-to-mesenchymal transition. Development 149. 10.1242/dev.200712. PMC9440755 35905012

[R23] Leathers TA, Rogers CD, 2023. Non-steroidal anti-inflammatory drugs and implications for the cyclooxygenase pathway in embryonic development. Am. J. Physiol.: Cell Physiol. 532–539. 10.1152/ajpcell.00430.2022. PMC9925163 36622071

[R24] Majumder M, Xin X, Liu L, Tutunea-Fatan E, Rodriguez-Torres M, Vincent K, Postovit L-M, Hess D, Lala PK, 2016. COX-2 induces breast cancer stem cells via EP4/PI3K/AKT/NOTCH/WNT Axis. Stem Cell. 34, 2290–2305. 10.1002/stem.2426. 27301070

[R25] Marshall EJ, Ramarapu R, Sandberg K, Kawashima M, Rogers CD, 2024. NSAID-mediated cyclooxygenase inhibition disrupts ectodermal derivative formation in axolotl embryos. bioRxiv 15, 37–48. 10.1016/j.diff.2025.100856 PMC12665286 40154219

[R26] Mo C, Zhao R, Vallejo J, Igwe O, Bonewald L, Wetmore L, Brotto M, 2015. Prostaglandin E2 promotes proliferation of skeletal muscle myoblasts via EP4 receptor activation. Cell Cycle 14, 1507–1516. 10.1080/15384101.2015.1026520. 25785867 PMC4615122

[R27] Morita I, Schindler M, Regier MK, Otto JC, Hori T, Dewitt DL, Smith WL, 1995. Different intracellular locations for prostaglandin endoperoxide H synthase-1 and-2. J. Biol. Chem. 270, 10902–10908. 10.1074/jbc.270.18.10902. 7738031

[R28] Murakami M, Nakashima K, Kamei D, Masuda S, Ishikawa Y, Ishii T, Ohmiya Y, Watanabe K, Kudo I, 2003. Cellular prostaglandin E2 production by membrane-bound prostaglandin E synthase-2 via both cyclooxygenases-1 and −2. J. Biol. Chem. 278, 37937–37947. 10.1074/jbc.M305108200. 12835322

[R29] Neil JR, Johnson KM, Nemenoff RA, Schiemann WP, 2008. Cox-2 inactivates Smad signaling and enhances EMT stimulated by TGF-β through a PGE2-dependent mechanisms. Carcinogenesis 29, 2227–2235. 10.1093/carcin/bgn202. 18725385 PMC2577139

[R30] Newton R, Kuitert LM, Slater DM, Adcock IM, Barnes PJ, 1996. Cytokine induction of cytosolic phospholipase A2 and cyclooxygenase-2 mRNA is suppressed by glucocorticoids in human epithelial cells. Life Sci. 60, 67–78. 10.1016/S0024-3205(96)00590-5. 8995534

[R31] Pajanoja C, Hsin J, Olinger B, Schiffmacher A, Abrams S, Dapkunas A, Zainul Z, Doyle A, Martin D, Kerosuo L, 2023. Maintenance of Pluripotency in the Entire Ectoderm Enables Neural Crest Formation. 10.1038/s41467-023-41384-6 PMC10518019 37741818

[R32] Park JY, Pillinger MH, Abramson SB, 2006. Prostaglandin E2 synthesis and secretion: the role of PGE2 synthases. Clin. Immunol. 119, 229–240. 10.1016/j.clim.2006.01.016. 16540375

[R33] Qiu X, Cheng JC, Chang HM, Leung PCK, 2014. COX2 and PGE2 mediate EGF-induced E-cadherin-independent human ovarian cancer cell invasion. Endocr. Relat. Cancer 21, 533–543. 10.1530/ERC-13-0450. 24969217

[R34] Ricciotti E, Fitzgerald GA, 2011. Prostaglandins and inflammation. Arterioscler. Thromb. Vasc. Biol. 31, 986–1000. 10.1161/ATVBAHA.110.207449. 21508345 PMC3081099

[R35] Rocca B, FitzGerald GA, 2002. Cyclooxygenases and prostaglandins: shaping up the immune response. Int. Immunopharm. 2, 603–630. 10.1016/S1567-5769(01)00204-1. 12013502

[R36] Satija R, Farrell JA, Gennert D, Schier AF, Regev A, 2015. Spatial reconstruction of single-cell gene expression data. Nat. Biotechnol. 33, 495–502. 10.1038/nbt.3192. 25867923 PMC4430369

[R37] Schill EM, Lake JI, Tusheva OA, Nagy N, Bery SK, Foster L, Avetisyan M, Johnson SL, Stenson WF, Goldstein AM, Heuckeroth RO, 2016. Ibuprofen slows migration and inhibits bowel colonization by enteric nervous system precursors in zebrafish, chick and mouse. Dev. Biol. 409, 473–488. 10.1016/j.ydbio.2015.09.023. 26586201 PMC4862364

[R38] Seo H, Choi Y, Shim J, Yoo I, Ka H, 2014. Comprehensive analysis of prostaglandin metabolic enzyme expression during pregnancy and the characterization of AKR1B1 as a prostaglandin F synthase at the maternal-conceptus interface in pigs. Biol. Reprod. 90, 1–13. 10.1095/biolreprod.113.114926. 24695626

[R39] Singh B, Berry J, Shoher A, Ramakrishnan V, Lucci A, 2005. COX-2 overexpression increases motility and invasion of breast cancer cells. Int. J. Oncol. 3, 118–129. 10.3892/ijo.26.5.1393. 15809733

[R40] Spear PC, Erickson CA, 2012. Interkinetic nuclear migration: a mysterious process in search of a function. Dev. Growth Differ. 54, 306–316. 10.1111/j.1440-169X.2012.01342.x. 22524603 PMC3357188

[R41] Speirs CK, Jernigan KK, Kim SH, Cha YI, Lin F, Sepich DS, DuBois RN, Lee E, Solnica-Krezel L, 2010. Prostaglandin Gβγ signaling stimulates gastrulation movements by limiting cell adhesion through Snai1a stabilization. Development 137, 1327–1337. 10.1242/dev.045971. 20332150 PMC2847468

[R42] Stanfield KM, Bell RR, Lisowski AR, English ML, Saldeen SS, Khan KNM, 2003. Expression of cyclooxygenase-2 in embryonic and fetal tissues during organogenesis and late pregnancy. Birth Defects Res A Clin Mol Teratol 67, 54–58. 12749384 10.1002/bdra.10032

[R43] Tombes RM, Borisy GG, 1995. The essential roles of calcium during mitosis. Adv. Mol. Cell. Biol. 13, 69–87. 10.1016/S1569-2558(08)60007-7.

[R44] Wang H, Wen Y, Mooney S, Behr B, Polan ML, 2002. Phospholipase A2 and cyclooxygenase gene expression in human preimplantation embryos. J. Clin. Endocrinol. Metab. 87, 2629–2634. 10.1210/jcem.87.6.8532. 12050227

[R45] Wilding M, 1996. Calcium and cell cycle control in early embryos. Zygote 4, 1–6. 10.1017/S0967199400002823. 8735364

[R46] Williams CS, DuBois RN, 1996. Prostaglandin endoperoxide synthase: why two isoforms? Am. J. Physiol. Gastrointest. Liver Physiol. 270. 10.1152/ajpgi.1996.270.3.g393. 8638704

[R47] Williams RM, Lukoseviciute M, Sauka-Spengler T, Bronner ME, 2022. Single-cell atlas of early chick development reveals gradual segregation of neural crest lineage from the neural plate border during neurulation. Elife 11, 1–21. 10.7554/eLife.74464. PMC8798042 35088714

[R48] Yazaki M, Kashiwagi K, Aritake K, Urade Y, Fujimori K, 2012. Rapid degradation of cyclooxygenase-1 and hematopoietic prostaglandin D synthase through ubiquitin-proteasome system in response to intracellular calcium level. Mol. Biol. Cell 23, 12–21. 10.1091/mbc.E11-07-0623. 22049022 PMC3248891

[R49] Yoon YH, Kim JY, Bae YC, Nam SW, Cho HJ, Lee S, Chung HY, Lee HS, Park MJ, 2018. Evaluation of the toxic effects of celecoxib on Xenopus embryo development. Biochem. Biophys. Res. Commun. 501, 329–335. 10.1016/j.bbrc.2018.03.002. 29505793

[R50] Zappia L, Oshlack A, 2018. Clustering trees: a visualization for evaluating clusterings at multiple resolutions. GigaScience 7, 1–9. 10.1093/gigascience/giy083. PMC6057528 30010766

[R51] Zha S, Yegnasubramanian V, Nelson WG, Isaacs WB, De Marzo AM, 2004. Cyclooxygenases in cancer: progress and perspective. Cancer Lett. 215, 1–20. 10.1016/j.canlet.2004.06.014. 15374627

[R52] Zidar N, Odar K, Glavac D, Jerse M, Zupanc T, Stajer D, 2009. Cyclooxygenase in normal human tissues - is COX-1 really a constitutive isoform, and COX-2 an inducible isoform? J. Cell Mol. Med. 13, 3753–3763. 10.1111/j.1582-4934.2008.00430.x. 18657230 PMC4516524

