# Supplementary material for: Spatiotemporal characterization of cyclooxygenase pathway enzymes during vertebrate embryonic development
Source: Dev Biol. Author manuscript; Available in PMC 2025 Mar 9. (PMC11890202; doi:10.1016/j.ydbio.2024.11.009)
Supplement: Supplemental figures [file NIHMS2055872-supplement-Supplemental_figures.pdf]

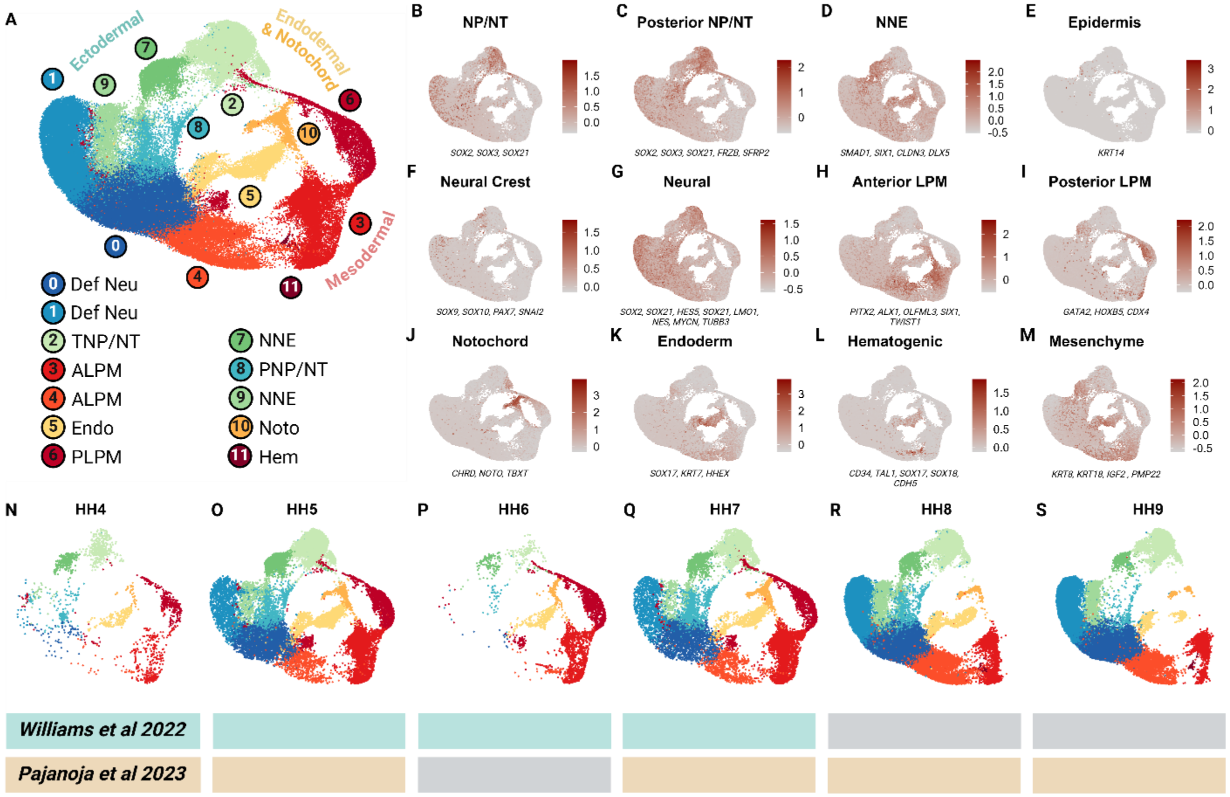

Supplemental Figure 1. Combined clustering of scRNA-seq datasets to identify cell types. Unsupervised clustering of publicly available scRNA-seq data of chick embryos between HH4 and HH9 demonstrates the presence of 9 major cell types across 11 clusters. (A) UMAP demonstrating the unsupervised clustering results of chick whole embryos. (B-M) Feature plot expression of cell type marker gene modules. Genes included in each module are listed under each plot. (N-S) UMAP split by stages. Blue colored blocks indicate cells sourced from Williams *et al* 2022 and orange colored blocks indicate cells sourced from Pajanoja *et al* 2023. Grey blocks indicate lack of data from the source publication at the specific age. Definitive Neural lineage, Def Neu; Transitional neural plate/ neural tube, TNP/NT; Anterior lateral plate mesoderm, ALPM; Endoderm, Endo; Posterior lateral plate mesoderm, PLPM; Non-neural ectoderm, NNE; Posterior neural plate/ neural tube, PNP/NT; Notochord, Noto; Hematogenic cells, Hem.

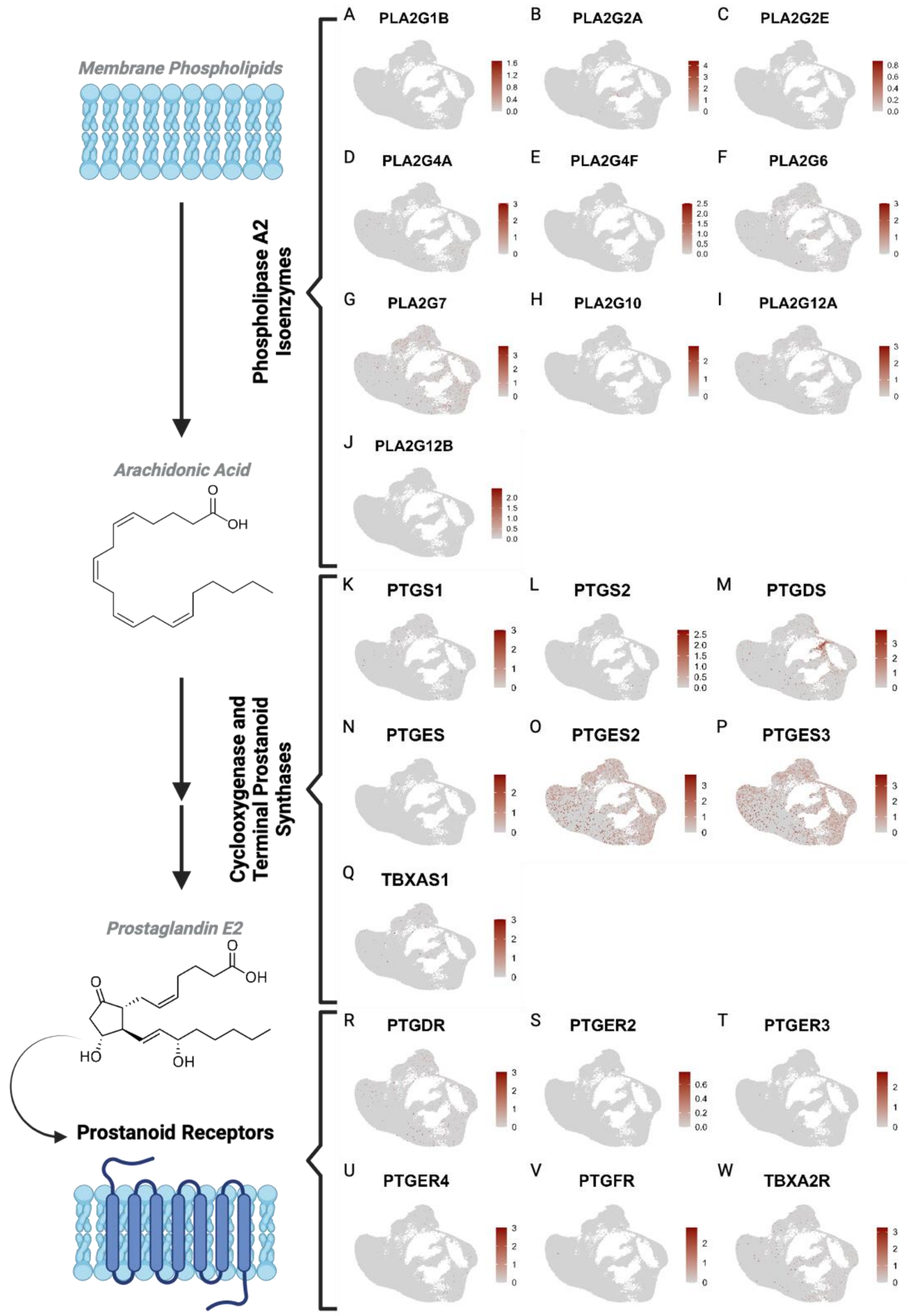

Supplemental Figure 2. Expression of COX pathway members in publicly available scRNA-seq data of chick embryos between HH4 and HH9. (A-J) Feature plot demonstrating expression of phospholipase A2 isoenzymes. (K-Q) Feature plot demonstrating expression of cyclooxygenases and terminal prostanoid synthases. (R-W) Feature plot demonstrating expression of prostanoid receptors.

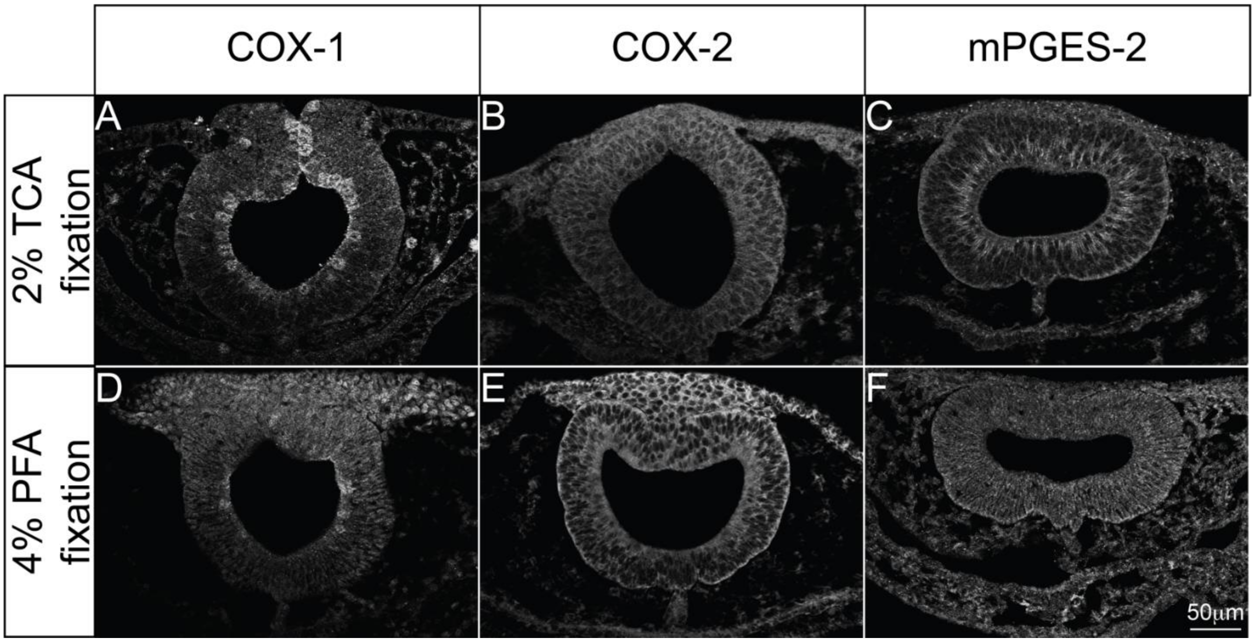

Supplemental Figure 3. The type of fixation applied to the embryo affects IHC visualization. (A-C) Transverse sections of whole chicken embryos fixed with 2% TCA for 1hr. (D-F) Using the same antibodies as (A-C), transverse sections of whole chicken embryos fixed with 4% PFA for 15-20min with a 1hr 4% PFA postfix after antibody incubation. Protein visualization of (A) COX-1 and (C) mPGES-2 appears more specific with 2% TCA fixation, while visualization of (B) COX-2 appears clearer with 4% PFA fixation. Scale bar for all in (F).

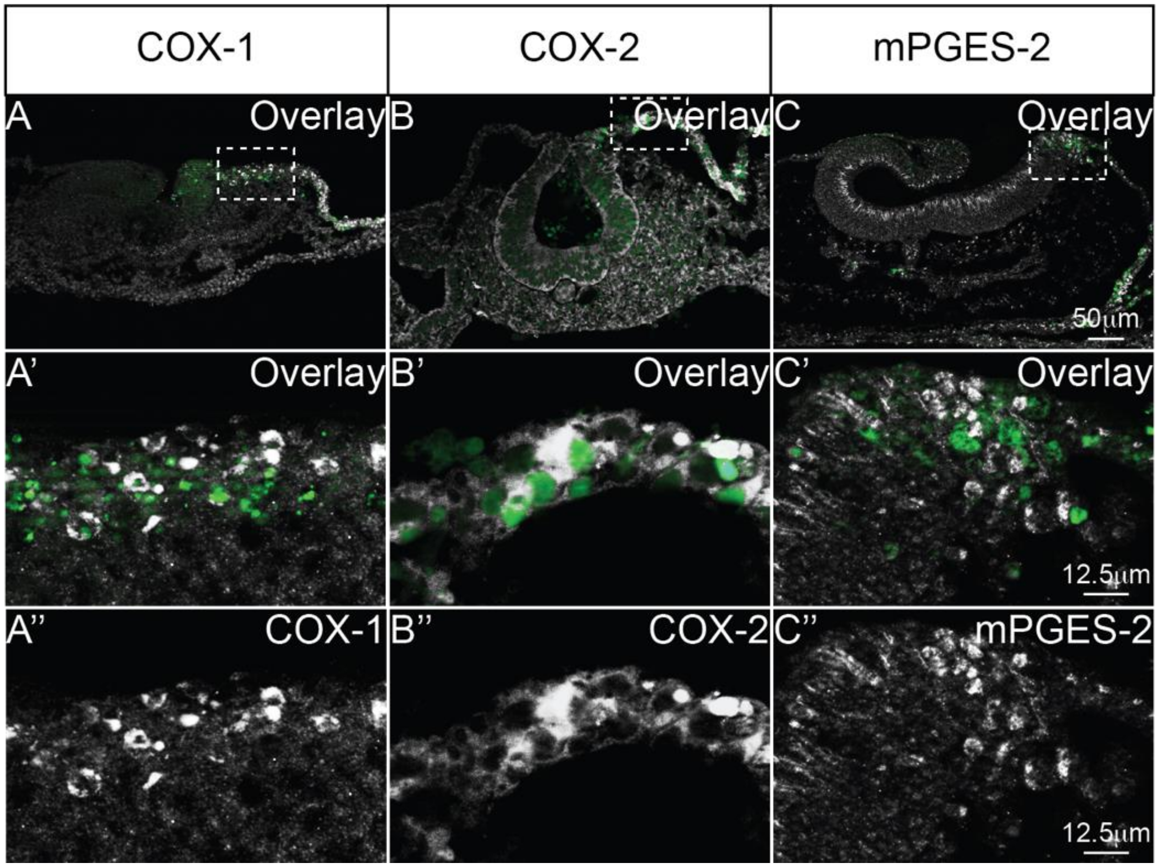

Supplemental Figure 4. Validation of antibodies used in study. (A-C) Transverse sections of whole chicken embryos unilaterally injected with full length vectors encoding (A-A'') COX-1, (B-B'') COX-2, or (C-C'') mPGES-2 for overexpression (green on right). (A'-C'') Zoom ins of region outlined in (A-C). (A'-C') Overlays show unilateral injection (green on right) along with antibodies against (A-A'') COX-1, (B-B'') COX-2, or (C-C'') mPGES-2 in white. Increase of antibody signal overlapping with injection signal after overexpression of the corresponding protein supports antibody specificity. (A-A'') COX-1 plasmid was co-injected with p53 morpholino to prevent cell death. Zoom-ins demonstrate cytoplasmic expression of all COX pathway proteins as shown in Fig. 3, 5, and 6.

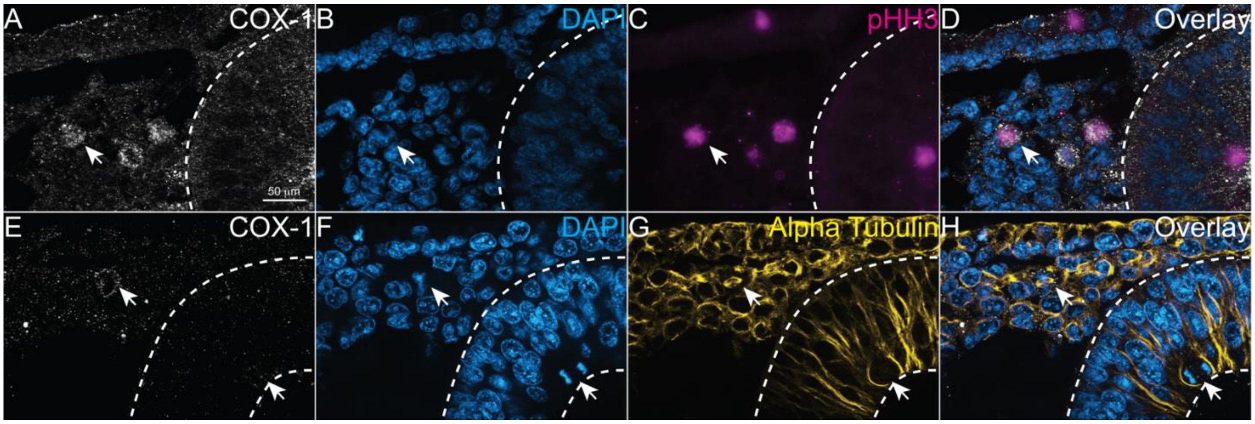

Supplemental Figure 5. COX-1 protein overlaps with mitosis markers in the chicken embryo. IHC with (B, F) the nuclear stain DAPI (blue) and antibodies against (A, E) the isoenzyme COX-1 (white), (G) the microtubule subunit Alpha Tubulin (yellow), and (C) the G2/M phase marker phosphohistone H3, pHH3

(magenta) in HH9 and HH10 chicken embryos shows that COX-1 is present in cells during mitosis. Overlays of all three channels shown in (D, H) and zoom-ins of cells indicated by arrows are shown in Fig. 4. Dashed outline indicates neural tube. Scale bar for all in (A).

Supplemental Table 1. Genes used for scRNA-seq analysis.

Gene Name	Ensembl ID	Reference Link	Type of Protein	Group
PLA2G1B	ENSGALG00010026939	http://useast.ensembl.org/Gallus_gallus/Gene/Summary?db=core;g=ENSGALG00010026939;r=15:9386184-9388894;t=ENSGALT00010065331	Phospholipase A2 isoenzymes	Group I
PLA2G2A	ENSGALG00010020777	http://useast.ensembl.org/Gallus_gallus/Gene/Summary?db=core;g=ENSGALG00010020777;r=21:4841318-4842594	Phospholipase A2 isoenzymes	Group II
PLA2G2E	ENSGALG00010020711	http://useast.ensembl.org/Gallus_gallus/Gene/Summary?db=core;g=ENSGALG00010020711;r=21:4832368-4834347	Phospholipase A2 isoenzymes	Group II
PLA2G3	ENSGALG00010022128	http://useast.ensembl.org/Gallus_gallus/Gene/Summary?db=core;g=ENSGALG00010022128;r=15:10005622-10008905	Phospholipase A2 isoenzymes	Group III
PLA2G4A	ENSGALG00010021027	http://useast.ensembl.org/Gallus_gallus/Gene/Summary?db=core;g=ENSGALG00010021027;r=8:10031242-10139649	Phospholipase A2 isoenzymes	Group IV
PLA2G4B	ENSGALG00010020250	http://useast.ensembl.org/Gallus_gallus/Gene/Summary?db=core;g=ENSGALG00010020250;r=5:24675349-24716529	Phospholipase A2 isoenzymes	Group IV
PLA2G4F	ENSGALG00010020568	http://useast.ensembl.org/Gallus_gallus/Gene/Summary?db=core;g=ENSGALG00010020568;r=5:25076338-25097909;t=ENSGALT00010049658	Phospholipase A2 isoenzymes	Group IV
PLA2G5	ENSGALG00010020717	http://useast.ensembl.org/Gallus_gallus/Gene/Summary?db=core;g=ENSGALG00010020717;r=21:4839236-4840674;t=ENSGALT00010050053	Phospholipase A2 isoenzymes	Group V
PLA2G6	ENSGALG00010013015	http://useast.ensembl.org/Gallus_gallus/Gene/Summary?db=core;g=ENSGALG00010013015;r=1:51018361-51038011	Phospholipase A2 isoenzymes	Group VI
PLA2G7	ENSGALG00010008293	http://useast.ensembl.org/Gallus_gallus/Gene/Summary?db=core;g=ENSGALG00010008293;r=3:109631987-109645042	Phospholipase A2 isoenzymes	Group VII
PLA2G10	ENSGALG00010020699	http://useast.ensembl.org/Gallus_gallus/Gene/Summary?db=core;g=ENSGALG00010020699;r=14:844247-873064	Phospholipase A2 isoenzymes	Group X

PLA2 G12A	ENSGALG00 010003850	http://useast.ensembl.org/Gallus_gallus/Gene/Summary?db=core;g=ENSGALG00010003850;r=4:57655495-57661579;t=ENSGALT00010008908	Phospholipase A2 isoenzymes	Group XII
PLA2 G12B	ENSGALG00 010021316	http://useast.ensembl.org/Gallus_gallus/Gene/Summary?db=core;g=ENSGALG00010021316;r=6:11939209-11950268;t=ENSGALT00010051652	Phospholipase A2 isoenzymes	Group XII
PTGS 1	ENSGALG00 010028372	http://useast.ensembl.org/Gallus_gallus/Gene/Summary?db=core;g=ENSGALG00010028372;r=17:9370346-9416687	Cyclooxygenase Isoenzymes	
PTGS 2	ENSGALG00 010021038	http://useast.ensembl.org/Gallus_gallus/Gene/Summary?db=core;g=ENSGALG00010021038;r=8:10138984-10147005	Cyclooxygenase Isoenzymes	
PTGD S	ENSGALG00 010028450	http://useast.ensembl.org/Gallus_gallus/Gene/Summary?db=core;g=ENSGALG00010028450;r=17:1184647-1187094	Terminal Prostanoid Synthases	
PTGE S	ENSGALG00 010028488	http://useast.ensembl.org/Gallus_gallus/Gene/Summary?db=core;g=ENSGALG00010028488;r=17:6278337-6294168;t=ENSGALT00010068980	Terminal Prostanoid Synthases	
PTGE S2	ENSGALG00 010028168	http://useast.ensembl.org/Gallus_gallus/Gene/Summary?db=core;g=ENSGALG00010028168;r=17:5779436-5784975;t=ENSGALT00010068251	Terminal Prostanoid Synthases	
PTGE S3	ENSGALG00 010025167	http://useast.ensembl.org/Gallus_gallus/Gene/Summary?db=core;g=ENSGALG00010025167;r=34:1543054-1550534	Terminal Prostanoid Synthases	
TBXA S1	ENSGALG00 010013152	http://useast.ensembl.org/Gallus_gallus/Gene/Summary?db=core;g=ENSGALG00010013152;r=1:55795267-56045421	Terminal Prostanoid Synthases	
PTGD R	ENSGALG00 010017946	http://useast.ensembl.org/Gallus_gallus/Gene/Summary?db=core;g=ENSGALG00010017946;r=5:57880272-57883325;t=ENSGALT00010043368	Prostanoid Receptors	
PTGE R2	ENSGALG00 010017954	http://useast.ensembl.org/Gallus_gallus/Gene/Summary?db=core;g=ENSGALG00010017954;r=5:57886644-57890428;t=ENSGALT00010043392	Prostanoid Receptors	
PTGE R3	ENSGALG00 010023902	http://useast.ensembl.org/Gallus_gallus/Gene/Summary?db=core;g=ENSGALG00010023902;r=8:28785032-28795733;t=ENSGALT00010058236	Prostanoid Receptors	
PTGE R4	ENSGALG00 010014350	http://useast.ensembl.org/Gallus_gallus/Gene/Summary?db=core;g=ENSGALG00010014350;r=Z:13372871-13383612;t=ENSGALT00010034553	Prostanoid	

			Receptors	
PTGFR	ENSGALG00010020693	http://useast.ensembl.org/Gallus_gallus/Gene/Summary?db=core;g=ENSGALG00010020693;r=8:18425758-18474762	Prostanoid Receptors	
TBXA2R	ENSGALG00010028120	http://useast.ensembl.org/Gallus_gallus/Gene/Summary?db=core;g=ENSGALG00010028120;r=28:1457445-1463230;t=ENSGALT00010068136	Prostanoid Receptors	
